# Plasma metabolic profiles predict future dementia and dementia subtypes: a prospective analysis of 274,160 participants

**DOI:** 10.1186/s13195-023-01379-3

**Published:** 2024-01-22

**Authors:** Yi-Xuan Qiang, Jia You, Xiao-Yu He, Yu Guo, Yue-Ting Deng, Pei-Yang Gao, Xin-Rui Wu, Jian-Feng Feng, Wei Cheng, Jin-Tai Yu

**Affiliations:** 1 Department of Neurology and National Center for Neurological Disorders, State Key Laboratory of Medical Neurobiology and MOE Frontiers Center for Brain Science, Huashan Hospital, Shanghai Medical College, Fudan University, 12Th Wulumuqi Zhong Road, Shanghai, 200040 China; 2 Institute of Science and Technology for Brain-Inspired Intelligence, Fudan University, Shanghai, 200433 China; 3 Department of Neurology, Qingdao Municipal Hospital, Qingdao University, Qingdao, 266011 China; 4 Key Laboratory of Computational Neuroscience and Brain-Inspired Intelligence, Ministry of Education, Fudan University, Shanghai, 200433 China; 5 Department of Computer Science, University of Warwick, Coventry, CV4 7AL UK

**Keywords:** Plasma, Metabolomics, Dementia, Alzheimer’s disease, Vascular dementia, Prediction

## Abstract

**Background:**

Blood-based biomarkers for dementia are gaining attention due to their non-invasive nature and feasibility in regular healthcare settings. Here, we explored the associations between 249 metabolites with all-cause dementia (ACD), Alzheimer’s disease (AD), and vascular dementia (VaD) and assessed their predictive potential.

**Methods:**

This study included 274,160 participants from the UK Biobank. Cox proportional hazard models were employed to investigate longitudinal associations between metabolites and dementia. The importance of these metabolites was quantified using machine learning algorithms, and a metabolic risk score (MetRS) was subsequently developed for each dementia type. We further investigated how MetRS stratified the risk of dementia onset and assessed its predictive performance, both alone and in combination with demographic and cognitive predictors.

**Results:**

During a median follow-up of 14.01 years, 5274 participants developed dementia. Of the 249 metabolites examined, 143 were significantly associated with incident ACD, 130 with AD, and 140 with VaD. Among metabolites significantly associated with dementia, lipoprotein lipid concentrations, linoleic acid, sphingomyelin, glucose, and branched-chain amino acids ranked top in importance. Individuals within the top tertile of MetRS faced a significantly greater risk of developing dementia than those in the lowest tertile. When MetRS was combined with demographic and cognitive predictors, the model yielded the area under the receiver operating characteristic curve (AUC) values of 0.857 for ACD, 0.861 for AD, and 0.873 for VaD.

**Conclusions:**

We conducted the largest metabolome investigation of dementia to date, for the first time revealed the metabolite importance ranking, and highlighted the contribution of plasma metabolites for dementia prediction.

**Supplementary Information:**

The online version contains supplementary material available at 10.1186/s13195-023-01379-3.

## Background

Dementia is one of the most common neurodegenerative disorders among the elderly, imposing considerable social and economic burden worldwide. As the initial symptoms of dementia are often subtle, by the time patients seek medical assistance, their brains have often undergone pathological changes that will persistently progress. Unfortunately, to date, the pharmacological treatment options for dementia are scarce. Therefore, identifying potential risk factors and developing predictive methods remain paramount in dementia research. Recently, blood-based biomarkers have received considerable attention due to their non-invasive nature and feasibility in regular healthcare settings.

Metabolites are small molecules that emerge from intricate cellular regulatory processes. Their concentrations in the blood provide insights into how different tissues produce and consume them, giving them the potential to serve as indicators of disease processes [1]. Recent research has indicated associations between blood metabolites and dementia risk [2–6]. However, most studies are limited by relatively small sample sizes and incomplete coverage of metabolites. The variation of metabolomic profiles among dementia subtypes is not well-investigated. Moreover, it remains uncertain which high-performing metabolites hold the most promise for predicting dementia. Prior research on the predictive performance of metabolites in dementia and cognitive impairment has yielded inconsistent results, with some showing positive outcomes [4, 5, 7, 8] and others not [9–11]. Moreover, previous studies have primarily focused on all-cause dementia (ACD) and Alzheimer’s disease (AD), but the potential of metabolites to predict vascular dementia (VaD) has not yet been investigated. Therefore, there is a clear need to conduct comprehensive, large-scale metabolomic studies that incorporate data on different dementia subtypes.

In this study, we leveraged metabolomic data from 274,160 individuals to perform an untargeted metabolomic investigation of dementia. Firstly, we comprehensively examined the associations between 249 metabolites and incident ACD, AD, and VaD. Then, we assessed the magnitude of the metabolites’ contribution to dementia prediction and analyzed how metabolites stratified the risk of dementia onset. Lastly, we explored the predictive performance of the top-ranked metabolites, whether in combination with demographic and cognitive indicators or not.

## Methods

### Study cohort

Our study extracted data from the UK Biobank (UKB), a prospective cohort comprising over 500,000 participants aged 40–69 years during recruitment (2006 to 2010). The participants were registered with the UK National Health Service, recruited from 22 assessment centers across the country, and monitored for an extended period. Among them, a random subset of 292,000 participants was characterized using a high-throughput 1H-nuclear magnetic resonance (NMR) metabolic biomarker platform developed by Nightingale Health Ltd. Ethics approval was obtained from the North West Multi-Centre Research Ethics Committee. All participants provided written informed consent. This study was conducted under UKB application number 19542.

### Plasma metabolite profiling

Our study utilized the metabolomic data from 292,000 UKB participants. In each plasma sample, 249 metabolic measures were quantified simultaneously, comprising 168 absolute levels and 81 derived ratios. The comprehensive biomarker profile included cholesterol, fatty acids, and low-molecular-weight metabolites, such as ketone bodies, amino acids, and glycolysis-related metabolites (Table S1). Technical details are available at (https://biobank.ndph.ox.ac.uk/showcase/ukb/docs/NMR_companion_phase2.pdf).

### Dementia outcome definition

Dementia diagnosis was ascertained by records from first occurrence reports (Fields 131,036–37, 130,836–43), algorithm definitions (Fields 375, 42,018–25), death registrations (Fields 40,001–02), and hospital inpatient data (Fields 41,270, 41,280). The diagnosis was based on the International Classification of Diseases 10th revision (ICD-10) codes: ACD (F00, F01, F02, F03, G30), AD (F00, G30), and VaD (F01), presented as a primary or secondary diagnosis in the health records or a potential cause of death in the death register (Table S2). Follow-up visits lasted from the initial assessment center attendance (Field 53) to the earliest date of dementia diagnosis, death, or the latest hospital inpatient data (March 2023), whichever occurred first.

### Associated metabolites identification and model development

We employed Cox proportional hazard (CPH) models to assess the associations between 249 metabolites and incident ACD, AD, and VaD. Potential confounders, including age, sex, educational years, and *APOE* ε4 carrier status, were adjusted as covariates. Multiple test corrections were applied using the false discovery rate (FDR) approach [12]. Statistical significance was set at *Q* value < 0.05. Metabolites that remain statistically significant in the Cox model after FDR adjustment proceed to the subsequent selection process.

To exploit the potential of metabolites as single-domain predictors for future ACD, AD, and VaD, we employed machine learning algorithms to further select essential metabolites and derived a metabolic risk score (MetRS) regarding each outcome. Specifically, we first estimated the importance of theses dementia-associated metabolites using information gain, an inherent statistic within the Light Gradient Boosting Machine (LightGBM) algorithm [13]. Next, we adopted a sequential selection approach employing LightGBM classifiers. These classifiers were iteratively refined by incorporating one metabolite at a time based on the order of pre-established importance. Iterations ceased when there was no significant improvement in the model’s area under the curve (AUC) performance, as indicated by three consecutive non-significant DeLong statistics [14]. The SHapley Additive exPlanations (SHAP) method was employed to visualize the metabolites’ risk or protective effect within the LightGBM classifier during MetRS development. Subsequently, we re-established a LightGBM classifier with the metabolites selected earlier and took its output risk probabilities as metabolic risk score (MetRS).

To analyze how metabolites stratify the risk of dementia onset, we employed several approaches. Accumulative incident rate curves were plotted on populations stratified based on tertiles of MetRS, and hazard ratios (HR) were reported between the top and bottom tertiles. Further subgroup analysis using CPH regressions was performed on dementia-related factors, such as age, sex, and *APOE* ε4 carrier status.

To explore metabolites’ predictive performance, we employed CPH models with three hierarchical predictor sets to calculate linear predictor (standardized log–log survival) of the 5-year, 10-year, and all incident dementia risks. Model 1 was developed based on MetRS alone. Subsequent models integrated MetRS with demographic indicators (age, sex, education, and *APOE* ε4 status) and cognitive tests (pairs matching time and reaction time) [15]. *P* value < 0.05 indicated statistical significance.

### Statistical analysis

Baseline characteristics of participants were compared between different dementia status, with continuous variables presented as median [interquartile range (IQR)] and categorical variables as number (percentage) in Table 1. A full comparison of 249 metabolites is available in Table S3.

**Table 1:** Baseline characteristics and distribution of the MetRS stratified by dementia status

Characteristics	Overall	Control	Incident dementia		Incident AD		Incident VaD	
	*N* = 274,160	*N* = 268,886	*N* = 5274	*P*	*N* = 2346	*P*	*N* = 1221	*P*
Age, year	58.00 [50.00, 63.00]	58.00 [50.00, 63.00]	65.00 [62.00, 68.00]	< 0.001	66.00 [63.00, 68.00]	< 0.001	66.00 [63.00, 68.00]	< 0.001
Female	147,969 (54.0)	145,485 (54.1)	2484 (47.1)	< 0.001	1224 (52.2)	0.064	480 (39.3)	< 0.001
White ethnicity	259,499 (95.1)	254,429 (95.0)	5070 (96.7)	< 0.001	2259 (96.9)	< 0.001	1182 (97.0)	0.008
Education, year	4.00 [3.00, 8.00]	4.00 [3.00, 8.00]	3.00 [2.00, 7.00]	< 0.001	3.00 [2.00, 7.00]	< 0.001	3.00 [2.00, 7.00]	< 0.001
*APOE* ε4, single-copy carriers	71,153 (26.2)	68,922 (25.8)	2231 (42.6)	< 0.001	1098 (47.3)	< 0.001	490 (40.4)	< 0.001
*APOE* ε4, double-copies carriers	6529 (2.4)	5984 (2.2)	545 (10.4)	< 0.001	322 (13.9)	< 0.001	113 (9.3)	< 0.001
Body mass index, Kg/m^2^	26.77 [24.17, 29.92]	26.76 [24.17, 29.91]	27.18 [24.52, 30.45]	< 0.001	26.99 [24.36, 30.00]	0.153	27.96 [25.19, 31.48]	< 0.001
Current smoker	28,853 (10.6)	28,320 (10.6)	533 (10.2)	0.386	201 (8.7)	0.003	142 (11.7)	0.214
Pairs matching time, second	188.50 [148.50, 244.50]	188.00 [148.50, 243.50]	226.50 [169.00, 314.50]	< 0.001	231.00 [172.50, 319.00]	< 0.001	229.50 [171.50, 325.00]	< 0.001
Reaction time, million second	538.00 [481.00, 610.00]	536.00 [480.00, 610.00]	586.00 [520.00, 672.00]	< 0.001	583.00 [520.00, 668.00]	< 0.001	597.00 [532.00, 686.25]	< 0.001
Standardized MetRS (ACD)	− 0.18 [− 0.71, 0.39]	− 0.18 [− 0.71, 0.38]	0.18 [− 0.30, 1.09]	< 0.001	0.14 [− 0.33, 0.99]	< 0.001	0.44 [− 0.19, 1.46]	< 0.001
Standardized MetRS (AD)	− 0.18 [− 0.63, 0.45]	− 0.19 [− 0.63, 0.45]	0.22 [− 0.35, 0.91]	< 0.001	0.21 [− 0.37, 0.87]	< 0.001	0.36 [− 0.28, 1.15]	< 0.001
Standardized MetRS (VaD)	− 0.32 [− 0.55, 0.25]	− 0.32 [− 0.55, 0.24]	0.05 [− 0.44, 0.84]	< 0.001	− 0.03 [− 0.46, 0.70]	< 0.001	0.27 [− 0.33, 1.16]	< 0.001
**Selected metabolites**								
His, mmol/L	0.06 [0.06, 0.07]	0.06 [0.06, 0.07]	0.06 [0.06, 0.07]	< 0.001	0.06 [0.06, 0.07]	< 0.001	0.06 [0.06, 0.07]	< 0.001
Leu, mmol/L	0.10 [0.08, 0.12]	0.10 [0.08, 0.12]	0.10 [0.08, 0.12]	< 0.001	0.10 [0.08, 0.12]	< 0.001	0.10 [0.08, 0.12]	0.808
Total BCAA, mmol/L	0.35 [0.31, 0.41]	0.35 [0.31, 0.41]	0.35 [0.30, 0.41]	< 0.001	0.34 [0.30, 0.40]	< 0.001	0.36 [0.30, 0.42]	0.261
Val, mmol/L	0.21 [0.18, 0.23]	0.21 [0.18, 0.23]	0.20 [0.18, 0.23]	< 0.001	0.20 [0.18, 0.23]	< 0.001	0.21 [0.18, 0.24]	0.142
LA%	29.10 [26.74, 31.24]	29.12 [26.76, 31.25]	28.06 [25.72, 30.38]	< 0.001	28.26 [26.12, 30.46]	< 0.001	27.34 [24.90, 29.70]	< 0.001
Omega-6/Omega-3	9.01 [7.16, 11.48]	9.02 [7.16, 11.49]	8.62 [6.85, 10.91]	< 0.001	8.54 [6.84, 10.66]	< 0.001	8.47 [6.71, 10.67]	< 0.001
LA, mmol/L	3.42 [2.99, 3.88]	3.42 [2.99, 3.88]	3.28 [2.79, 3.80]	< 0.001	3.34 [2.85, 3.85]	< 0.001	3.18 [2.70, 3.71]	< 0.001
Glu, mmol/L	3.56 [3.12, 4.04]	3.56 [3.11, 4.03]	3.73 [3.26, 4.30]	< 0.001	3.70 [3.26, 4.27]	< 0.001	3.81 [3.32, 4.45]	< 0.001
IDL-C, mmol/L	0.83 [0.69, 0.98]	0.83 [0.69, 0.98]	0.79 [0.62, 0.96]	< 0.001	0.83 [0.64, 0.99]	0.003	0.73 [0.58, 0.92]	< 0.001
IDL-P, mmol/L	0.00 [0.00, 0.00]	0.00 [0.00, 0.00]	0.00 [0.00, 0.00]	< 0.001	0.00 [0.00, 0.00]	0.003	0.00 [0.00, 0.00]	< 0.001
SM, mmol/L	0.45 [0.40, 0.50]	0.45 [0.40, 0.50]	0.44 [0.38, 0.50]	< 0.001	0.45 [0.39, 0.51]	0.41	0.42 [0.37, 0.48]	< 0.001
IDL-CE%	50.30 [48.64, 51.66]	50.31 [48.66, 51.67]	49.62 [47.70, 51.21]	< 0.001	49.82 [48.14, 51.35]	< 0.001	49.14 [47.11, 50.84]	< 0.001
IDL-FC%	17.81 [17.07, 18.47]	17.81 [17.07, 18.47]	17.77 [16.96, 18.46]	0.002	17.83 [17.02, 18.55]	0.62	17.71 [16.80, 18.43]	< 0.001
L-HDL-TG%	4.78 [3.37, 6.94]	4.77 [3.37, 6.94]	5.07 [3.55, 7.29]	< 0.001	4.96 [3.48, 7.16]	0.001	5.42 [3.68, 7.72]	< 0.001
S-LDL-CE%	45.90 [44.55, 47.20]	45.90 [44.55, 47.20]	45.62 [44.28, 46.98]	< 0.001	45.70 [44.32, 47.01]	< 0.001	45.50 [44.17, 46.84]	< 0.001
S-LDL-C%	63.50 [62.18, 64.57]	63.51 [62.20, 64.58]	63.00 [61.48, 64.25]	< 0.001	63.19 [61.76, 64.38]	< 0.001	62.60 [60.99, 64.02]	< 0.001
S-LDL-PL%	31.19 [30.12, 32.33]	31.18 [30.12, 32.32]	31.41 [30.34, 32.54]	< 0.001	31.37 [30.30, 32.48]	< 0.001	31.48 [30.39, 32.59]	< 0.001

Values are reported as median [interquartile range] for continuous variables or numbers (percentage) for categorical variables. *P*-values are derived using either Student’s *t*-test or chi-square test. Metabolites selected from three dementia type models were shown here, see all 249 metabolites baseline characters in Table S2

*Abbreviations*: *His*, histidine; *Leu*, leucine; *BCAA*, branched-chain amino acids; *Val*, valine; *LA*, linoleic acid; *Glu*, glucose; *SM*, sphingomyelins; *C*, cholesterol; *CE*, cholesteryl esters; *FC*, free cholesterol; *P*, particles; *PL*, phospholipids; *TG*, triglycerides

For the main analysis, we removed metabolite outliers defined as values beyond four IQRs from the median. We then adjusted natural-log transformed metabolite levels for the NMR spectrometer (Field 23,650) by fitting a linear regression model and scaled the residuals for downstream analysis [16]. Individuals with over 20% missing metabolite values were excluded from the association analysis and prediction modeling. As for the modeling of the MetRS, we did not address missing data since the LightGBM model, being a missingness tolerant algorithm, can automatically handle it during model training and prediction.

To maximize the generalizability and transferability of MetRS and its downstream survival analysis, we employed a nested cross-validation scheme by spatially partitioning the UKB into ten folds based on the geographical locations of assessment centers (Table S4). Within each iteration, a LightGBM classifier was trained on nine folds and tested on the remaining one. This process was iterated until each fold served as both a training and testing set. The survival prediction modeling followed the same partitions. Then, all predictions from the testing sets were aggregated and evaluated using a bootstrap method over 2000 iterations. Notably, hyperparameter tuning was performed within the training set (nine folds of data) by randomly splitting the data into 80% and 20% for model training and validation. The testing set remained untouched within each iteration, reserved exclusively for model evaluations.

Association analysis and survival prediction analysis using CPH regressions were performed with survival, survminer, and pROC package in R (v4.2.0). MetRS development using LightGBM and its SHAP visualization were implemented through LightGBM (v3.3.2) and Shap (v0.40.0) under the Python (v3.9) environment.

## Results

### Cohort characteristics

After excluding participants with dementia at baseline, we included 274,160 individuals for metabolomic investigation. Participants’ characteristics stratified by dementia status were presented in Table 1. Overall, the median age was 58 (IQR: 50–63) years; 147,969 (54%) were females, and 259,499 (95.1%) were of white ancestry. Compared with the control group, the ACD, AD, and VaD groups demonstrated an older age, a greater proportion of males and *APOE* ε4 carriers, lower educational attainment, prolonged pairs matching time and reaction time, along with an elevated MetRS. During a median follow-up of 14.01 years (IQR: 13.24–14.73 years), 5274 (1.92%) participants developed dementia, of which 2346 were AD and 1221 were VaD.

### Identifying metabolites associated with incident dementia

**Fig. 1:**
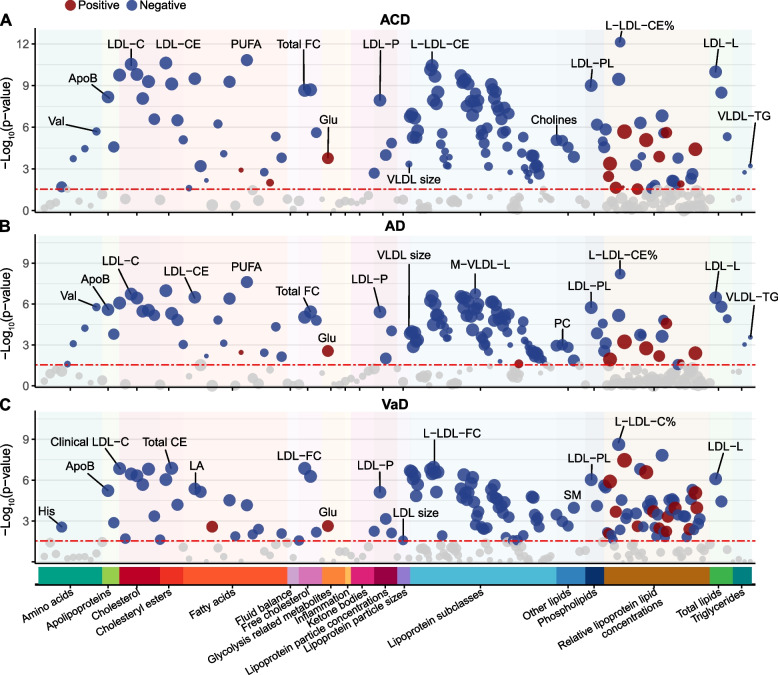
Dementia-associated metabolites in the association analysis. Significant associations (FDR–corrected *Q* value < 0.05) are shown, with red and blue colors respectively indicate the positive and negative effect directions and circle size proportional to the effect size. The most significant association for each metabolite group is labeled. Abbreviations: ACD, all-cause dementia; AD, Alzheimer’s disease; ApoB, apolipoprotein B; CE, cholesteryl esters; FC, free cholesterol; Glu, glucose; His, histidine; LA, linoleic acid; LDL, low-density lipoprotein; L-LDL, large LDL; LDL-L, total lipids in LDL; LDL-P, concentration of LDL particles; M-VLDL-L, total lipids in medium VLDL; PC, phosphatidylcholines; PL, phospholipids; PUFA, polyunsaturated fatty acids; SM, sphingomyelin; VaD, vascular dementia; Val, valine; VLDL-TG, triglycerides in VLD

Of the 249 metabolites examined, 143 were significantly associated with incident ACD, 130 with AD, and 140 with VaD (Fig. 1). HR, 95% confidence interval (CI), *P* value, and *Q* value were reported in Table S5. Generally, associations had larger effect sizes for VaD than ACD and AD, consistent with prior studies highlighting a stronger cardiometabolic influence on VaD [17].

The strongest positive metabolite-dementia association was observed with the ratio of triglycerides to total lipids in large low-density lipoprotein percentage (L-LDL-TG%) (HR = 1.10, 95% CI 1.06–1.14, *Q* = 8.44 × 10^−6^), notably more pronounced in VaD (HR = 1.31, 95% CI 1.19–1.45, *Q* = 2.90 × 10^−6^). Conversely, the ratio of cholesteryl esters to total lipids in large LDL percentage demonstrated the strongest negative association with dementia (HR = 0.87, 95% CI 0.84–0.90, *Q* = 1.90 × 10^−10^) and specifically with AD (HR = 0.88, 95% CI 0.84–0.92, *Q* = 1.57 × 10^−6^). This highlighted the close relationship between dementia and lipoprotein lipid ratios. In general, the ratios of cholesterol, free cholesterol, and cholesterol esters to total lipids showed a protective effect against dementia, while the ratios of triglycerides and phospholipids to total lipids were predominantly harmful. In terms of absolute lipoprotein lipid concentrations, almost all significant indicators showed a protective effect against dementia. Small high-density lipoprotein (HDL) was significantly associated with dementia, whereas large, medium, and very large HDL were not. Moreover, LDL, intermediate-density lipoprotein (IDL), and various sizes of very low-density lipoprotein (VLDL) were broadly associated with dementia risk.

Regarding fatty acids, the ω6 polyunsaturated fatty acids (PUFA) subgroup was significantly associated with dementia. Linoleic acid (LA), in particular, demonstrated negative associations (HR range: 0.79 to 0.89, *Q* range: 6.42 × 10^−9^ to 3.11 × 10^−5^), while LA to total fatty acids percentage (LA%) showed negative associations with ACD and VaD (HR range: 0.81 to 0.94, *Q* range: 4.63 × 10^−5^ to 0.001). Saturated fatty acids, monounsaturated fatty acids, and ω3 PUFA subgroup, including docosahexaenoic acid, demonstrated significant associations with ACD and AD. Additionally, the ratio of ω6 PUFA to ω3 PUFA was positively associated with ACD and AD (HR = 1.07, *Q* range: 0.003 to 0.008). And regarding other lipids, sphingomyelin, total choline, phosphatidylcholines, and phosphoglycerides exhibited protective effects against dementia (HR range: 0.81 to 0.94, *Q* range: 8.31 × 10^−6^ to 0.03).

In the glycolysis category, glucose was the only metabolite significantly associated with dementia incidence: ACD (HR = 1.08, 95% CI 1.04–1.12, *Q* = 4 × 10^−4^), AD (HR = 1.07, 95% CI 1.01–1.12, *Q* = 0.007), and VaD (HR = 1.18, 95% CI 1.06–1.31, *Q* = 0.006).

As for amino acids, branched-chain amino acids (BCAAs) demonstrated strong associations with ACD and AD (HR range: 0.89 to 0.95, *Q* range: 8.31 × 10^−6^ to 0.049). Specifically, valine, leucine, and total BCAAs were significantly associated with ACD and AD, while isoleucine was exclusively associated with AD. Notably, histidine was the only amino acid significantly associated with VaD risk (HR = 0.85, 95% CI 0.77–0.95, *Q* = 0.007).

Subgroup analysis produced largely consistent results (Table S6-S8). Importantly, BCAAs were associated with ACD and AD exclusively in females and *APOE* ε4 carriers. The associations between lipoprotein lipid concentrations and fatty acids with dementia were more pronounced in males and the elderly.

### Metabolite importance ranking and MetRS calculation

**Fig. 2:**
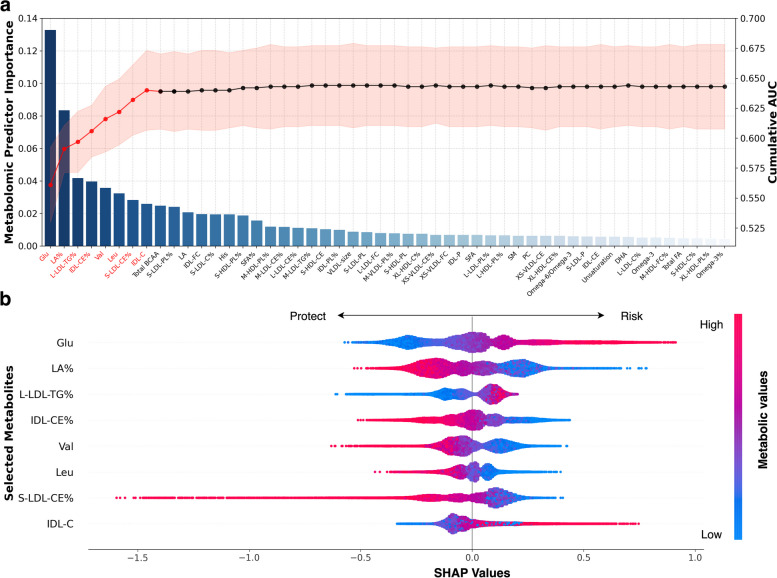
Metabolites importance ranking and SHAP visualization of modeling based on incident ACD populations. **A** Metabolites that survived FDR corrections in the association analysis further underwent sequential forward selection. The bar chart illustrates the importance of metabolites (left axis), ranked in ascending order. The line chart depicts cumulative area under the curve (AUC) values (right axis) as metabolites are included in successive iterations. The metabolites ultimately selected for MetRS calculation are highlighted in red. **B** Individual SHAP values of the selected metabolites are ranked according to their contributions. The *x*-axis represents the scale of the SHAP values for every metabolite, indicating their contribution to the prediction. The color range corresponds to each metabolic value, from blue (low value) to red (high value). Abbreviations: AUC, area under the curve; SHAP, SHapley Additive exPlanations

Among the 143 dementia-associated metabolites, we identified the eight most important ones for ACD prediction using the sequential forward selection strategy: glucose, LA%, L-LDL-TG%, cholesteryl esters to total lipids in IDL percentage (IDL-CE%), valine, leucine, cholesteryl esters to total lipids in small LDL percentage (S-LDL-CE%), and cholesterol in IDL (Fig. 2A). The line chart demonstrated a sharp rise in predictive performance when modeling with key metabolites, with the curve plateauing as more metabolites were incorporated. We further established a MetRS for ACD based on the selected metabolites.

We employed the SHAP summary plot to visualize each metabolite’s influence in the model (Fig. 2B). Specifically, glucose ranked highest in metabolite importance ordering. Participants with higher glucose levels (colored in red) were more likely to develop dementia (right side), whereas those with lower levels (blue) tended to remain healthy (left). LA% ranked second, with lower values enhancing predictions and higher values decreasing them. L-LDL-TG% only offered protection in the lower percentiles, with no obvious risk effect at high values. Similar explanations were given for the remaining metabolites.

For AD and VaD, eight and nine metabolites were respectively selected to construct their MetRS (Fig. S1, Fig. S2, Table S9). Generally, LA or LA%, glucose, and L-LDL-TG% were important in predicting all three types of dementia. Specifically, total BCAAs and sphingomyelin were pivotal for AD prediction, while histidine was crucial for VaD prediction.

### MetRS stratifies the risk of dementia onset

**Fig. 3:**
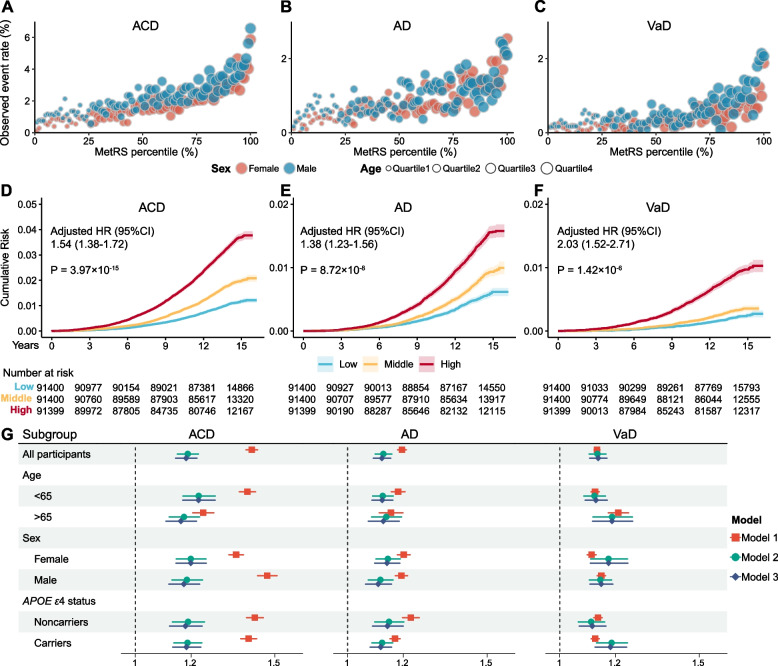
MetRS stratifies the risk of dementia onset. **A**–**C** Observed event rate for incident ACD, AD and VaD, plotted against MetRS percentiles over the entire study population. Blue dots represent males and red dots represent females. The size of each dot is proportional to age. **D**–**F** Cumulative risk over the observation time for incident ACD, AD and VaD, stratified by MetRS tertiles (light blue, bottom tertile; blue, median tertile; dark blue, top tertile). The shaded area indicates the 95% CI of the survival curves. **G** Regression results of MetRS and dementia outcomes in all participants and subgroups. Model 1, MetRS; Model2, MetRS + demographic indicators; Model 3, MetRS + demographic indicators + cognitive indicators. Abbreviations: ACD, all-cause dementia; AD, Alzheimer’s disease; VaD, vascular dementia

Next, we explored how MetRS stratifies the risk of dementia onset. Participants with a higher percentile of MetRS at baseline exhibited elevated event rates than those in lower percentiles, concurrently with an increase in age. Within the same percentile of MetRS, males displayed a higher risk of dementia compared to females (Fig. 3A–C). The Kaplan–Meier survival curves showed different cumulative risk trajectories for each tertile stratified by MetRS. Individuals within the top tertile of MetRS faced a greater risk of developing dementia than those in the lowest tertiles (HR range: 1.38 to 2.03, *P* range: 3.97 × 10^−15^ to 1.42 × 10^−6^, Fig. 3D–F). We also evaluated the effect of MetRS on dementia incidence in CPH models. An increase of one standard deviation (s.d.) in MetRS significantly increased dementia risk (HR range: 1.13 to 1.42, *P* range: 0 to 1.31 × 10^−151^). The inclusion of demographic or cognitive indicators in adjusted models did not substantially alter effect estimates. These results were generally replicated across different subgroups (Fig. 3G, Table S10).

### Predictive performance of MetRS

**Fig. 4:**
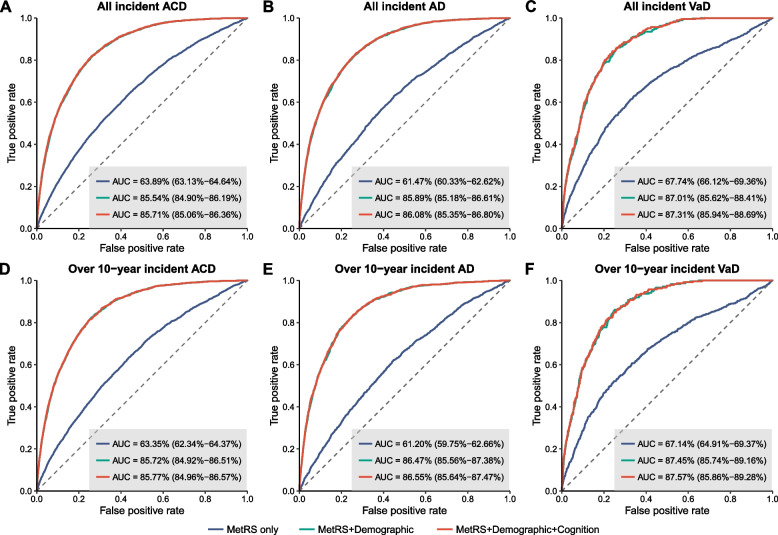
Prediction of incident ACD, AD, and VaD. Receiver operating characteristic (ROC) curves show the predictive performance of MetRS, either alone or in combination with demographic and cognitive indicators, for all incident cases (**A**–**C**), as well as for over 10-year incident cases (**D**–**F**) of ACD, AD, and VaD. The dotted line indicates an AUC of 0.50 for comparison. AUC estimates and 95% CIs are shown in Table S10. Abbreviations: AUC, area under the curve; ACD, all-cause dementia; AD, Alzheimer’s disease; VaD, vascular dementia

We then assessed the predictive performance of MetRS for future dementia. For all incident ACD, MetRS alone demonstrated an AUC of 0.639 (95% CI 0.631–0.646). When combined with demographic predictors, the AUC of the model elevated to 0.855 (95% CI 0.849–0.862). Further adding cognitive indicators slightly increased the AUC to 0.857 (95% CI 0.851–0.864). Patterns of model performance for predicting AD and VaD were similar. The full model combining MetRS with demographic and cognitive indicators provided optimal predictive performance for both AD (AUC = 0.861, 95% CI 0.854–0.868) and VaD (AUC = 0.873, 95% CI 0.859–0.887). Utilizing these models for predicting 5-year, 10-year, and over 10-year probability of dementia, we consistently obtained robust results (Fig. 4, Fig. S3, Table S11).

## Discussion

Utilizing data from 274,160 UKB participants, we compared the NMR-derived metabolic profiles among three types of dementia; for the first time, we revealed the importance ranking of 249 metabolites and assessed their potential in predicting dementia onset. Our results not only validated the extensive associations between plasma metabolites and dementia but also indicated that integrating these metabolites into dementia prediction models could refine classifications for populations at risk and contribute to predictive performance.

Lipoproteins are heterogeneous classes of particles. Importantly, small HDL and its lipid components exhibit broad associations with AD, whereas medium and large HDL do not. This aligns with the knowledge that small HDL, potentially the only lipoprotein that can penetrate the blood–brain barrier (BBB) [18], plays essential roles in lipid metabolism, inflammation, and anti-oxidation within the brain [5, 19, 20]. The relationship between VLDL, LDL, and IDL with dementia is less direct since these lipoproteins cannot penetrate the BBB. Of particular note, the ratios of cholesterol, free cholesterol, and cholesterol esters to total lipids appeared to have a protective effect against the studied dementia types. Conversely, the ratios of triglycerides and phospholipids to total lipids seemed to be primarily detrimental. The ratios L-LDL-TG%, S-LDL-CE%, and IDL-CE%, which have rarely been emphasized previously, show significant importance in metabolite ranking for dementia prediction in our study. An adequate explanation of these findings is yet to be realized.

Linoleic acid is the most abundant PUFA in human diets. It serves as a structural component of cell membranes and the precursor to the ω6 PUFA family [21]. Limited studies examining the associations between LA and ω6 PUFAs with dementia yielded inconsistent results. Our findings agree with several studies that higher plasma levels of LA and ω6 PUFAs are linked to reduced risks of developing dementia [22, 23]. Consistent with the known pro-inflammatory, pro-atherogenic, and pro-thrombotic properties of ω6 PUFAs in contrast with ω3 PUFAs [21, 24], our findings also suggest that elevated ω6 to ω3 ratios correlate with a heightened dementia risk [25]. Future research is still needed to validate these observations.

Sphingomyelin is an important lipid component of the myelin sheath, facilitating electrical impulse conduction along axons. In the central nervous system, sphingomyelin may influence amyloid precursor protein processing and neuronal excitability, thus impacting AD progression [26, 27]. While previous research has yielded mixed results, our findings support that elevated sphingomyelin levels correlate with a reduced dementia risk [4, 27, 28]. Moreover, our study further advances the understanding of sphingomyelin’s potential predictive role in AD.

Glucose was positively associated with all three types of dementia and consistently ranked in the top three in metabolite importance ordering, uncovering its pivotal role in dementia prediction. Elevated glucose is an established risk factor for dementia in the elderly, even among people without diabetes [4, 6, 29]. Higher levels of glucose have deleterious effects on the aging brain through several mechanisms, including glucose neurotoxicity [30–32], insulin resistance [33–35], cardiovascular events [36, 37], and inflammation [38, 39].

BCAAs, including isoleucine, leucine, and valine, are essential amino acids that our body cannot produce and must acquire from dietary sources. Their circulating levels can be influenced by genetic background and metabolic disturbances [40]. BCAAs can penetrate the BBB, serving both as an energy source for mitochondria and a nitrogen donor for neurotransmitters, highlighting their importance in preserving brain health. Our findings align with prior research suggesting a negative association between BCAAs and the onset of ACD and AD [41–45]. We also identified a protective effect of histidine on incident VaD.

By integrating key metabolites identified in our association analysis and importance ranking, we developed a MetRS for each dementia outcome. Our findings underscore the potential of plasma metabolites to reclassify at-risk populations, setting the stage for proactive interventions to reduce dementia incidence in the future. Moreover, we found that the incorporation of MetRS with demographic and cognitive indicators achieved a satisfactory AUC of 0.86, although the absolute enhancement was not substantial. This can be attributed to the fact that traditional risk factors, such as hypertension and diabetes, already accounted for some of the metabolic changes observed in the development of dementia. Further investigation is needed to elucidate this. Furthermore, for the first time, we have unveiled the contribution of metabolites in predicting VaD.

Our study has several notable strengths. Firstly, unlike traditional methods, metabolomics allows for simultaneous investigation of hundreds of metabolites in blood, providing a comprehensive metabolic profile in dementia patients. Secondly, we leveraged the largest plasma metabolomics dataset available to date, comprising 274,160 participants with a median follow-up of 14.01 years. Thirdly, we ranked the metabolites by importance using a machine learning approach and identified markers undervalued in previous research. Furthermore, we compared the metabolic profiles among ACD, AD, and VaD and established a MetRS for each.

Before application in routine care, there are still challenges to be addressed. Firstly, the present coverage of metabolites is lipid-focused, which needs to be expanded by additional techniques. Secondly, given that UKB participants are predominantly younger, healthier, better educated, and of European ancestry, our findings require further validation in other populations. Thirdly, we cannot conclude any cause-effect relationship due to the observational nature of our study. In addition, while we identified potential dementia cases through hospital admissions, death registers, and primary care, our reliance on electronic health records to ascertain dementia might omit subclinical cases, thereby undermining the sensitivity of our prediction. Finally, the direct comparison between metabolomics parameters and neurodegenerative parameters in their ability to predict dementia remains unexplored, for which further studies are needed.

## Conclusions

Taken together, we are the largest metabolomic study conducted to date. The population-based cohort, the growing accessibility of cutting-edge metabolic biomarkers, and the refinement of statistical methodologies for intricate prediction modeling have collectively advanced our understanding of dementia. The MetRS, being non-invasive, avoids the potential risks of lumbar punctures and radiological exams. It can be incorporated into routine check-ups that involve blood tests more easily. Our findings may improve the effective screening of at-risk populations and represent a target of choice for the primary prevention of dementia in the near future. represent a target of choice for the primary prevention of dementia.

### Supplementary Information

**Additional file 1: Table S1. **UKB Nightingale metabolite measurements and clinical parameters information. **Table S2.** Detailed dementia outcome definition. **Table S3.** All 249 metabolites baseline characters of the study participants by dementia case-control status. **Table S4.** Comparison of covariate distributions for 10 regions. **Table S5.** Results of associations between plasma metabolites and incident dementia. **Table S6.** Results of associations between plasma metabolites and incident dementia stratified by sex. **Table S7.** Results of associations between plasma metabolites and incident dementia stratified by age. **Table S8.** Results of associations between plasma metabolites and incident dementia stratified by APOE4 carrier status. **Table S9.** Results of metabolites importance ranking. **Table S10.** The hazard ratios of MetRS in the full cohort and subgroups. **Table S11.** Discriminative performance of CPH models.

**Additional file 2:  Supplemental Fig. 1.** Selected metabolites to construct the MetRS of AD

**Additional file 3: Supplemental Fig. 2.** Selected metabolites to construct the MetRS of VaD

**Additional file 4: Supplemental Fig. 3.** Results from utilizing the models for predicting 5-year, 10-year, and over 10-year probability of dementia

## Data Availability

The data supporting this study’s findings are available from the UK Biobank project site, subject to registration and application process. Further details can be found at https://www.ukbiobank.ac.uk.

## Abbreviations

ACD: All-cause dementia
AD: Alzheimer’s disease
AUC: Area under the curve
BBB: Blood-brain barrier
BCAAs: Branched-chain amino acids
CI: Confidence interval
CPH: Cox proportional hazard
FDR: False discovery rate
HDL: High-density lipoprotein
HR: Hazard ratios
IDL: Intermediate-density lipoprotein
IDL-CE%: Cholesteryl esters to total lipids in IDL percentage
IQR: Interquartile range
LA: Linoleic acid
LA%: LA to total fatty acids percentage
LightGBM: Light Gradient Boosting Machine
L-LDL-TG%: Triglycerides to total lipids in large low-density lipoprotein percentage
MetRS: Metabolic risk scores
NMR: Nuclear magnetic resonance
PUFA: Polyunsaturated fatty acids
S-LDL-CE%: Cholesteryl esters to total lipids in small LDL percentage
SHAP: SHapley Additive exPlanations
UKB: UK Biobank
VaD: Vascular dementia
VLDL: Very low-density lipoprotein
