# Supplementary figures and images for: Plasma metabolic profiles predict future dementia and dementia subtypes: a prospective analysis of 274,160 participants
Source: Alzheimers Res Ther. 2024 Jan 22;16:16. doi: 10.1186/s13195-023-01379-3 (PMC10802055; doi:10.1186/s13195-023-01379-3)

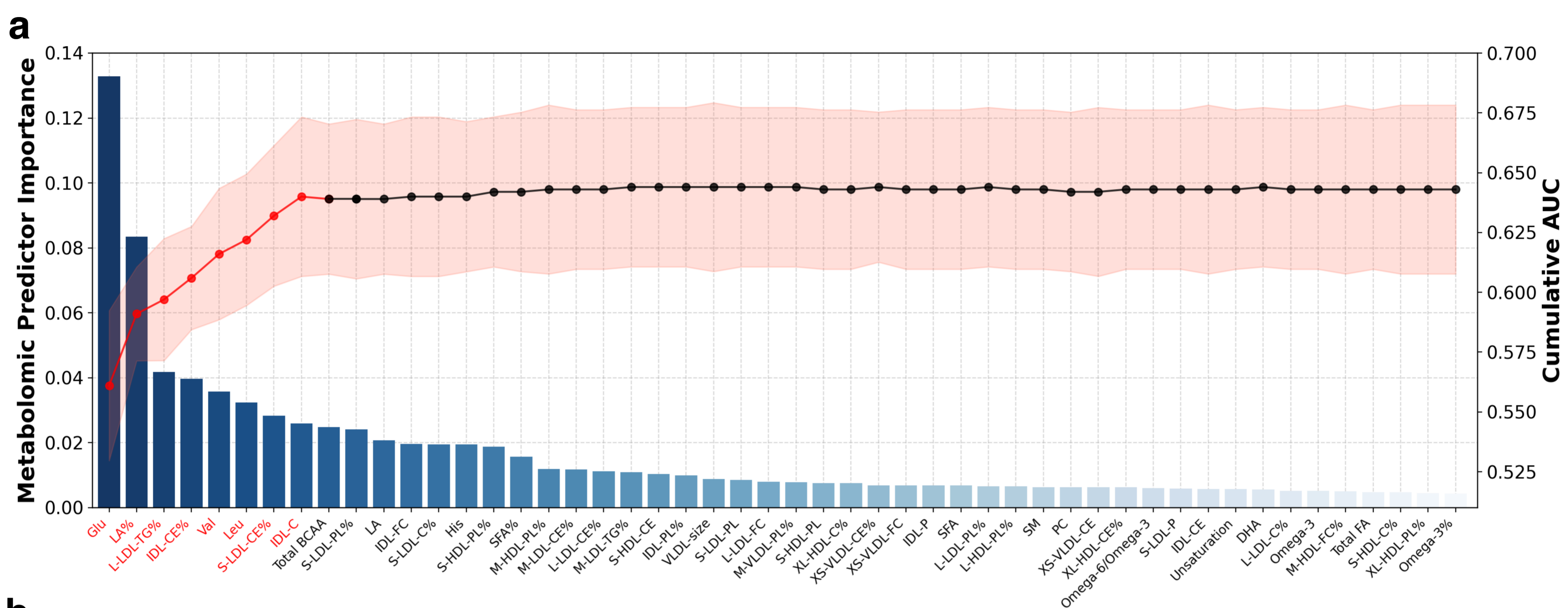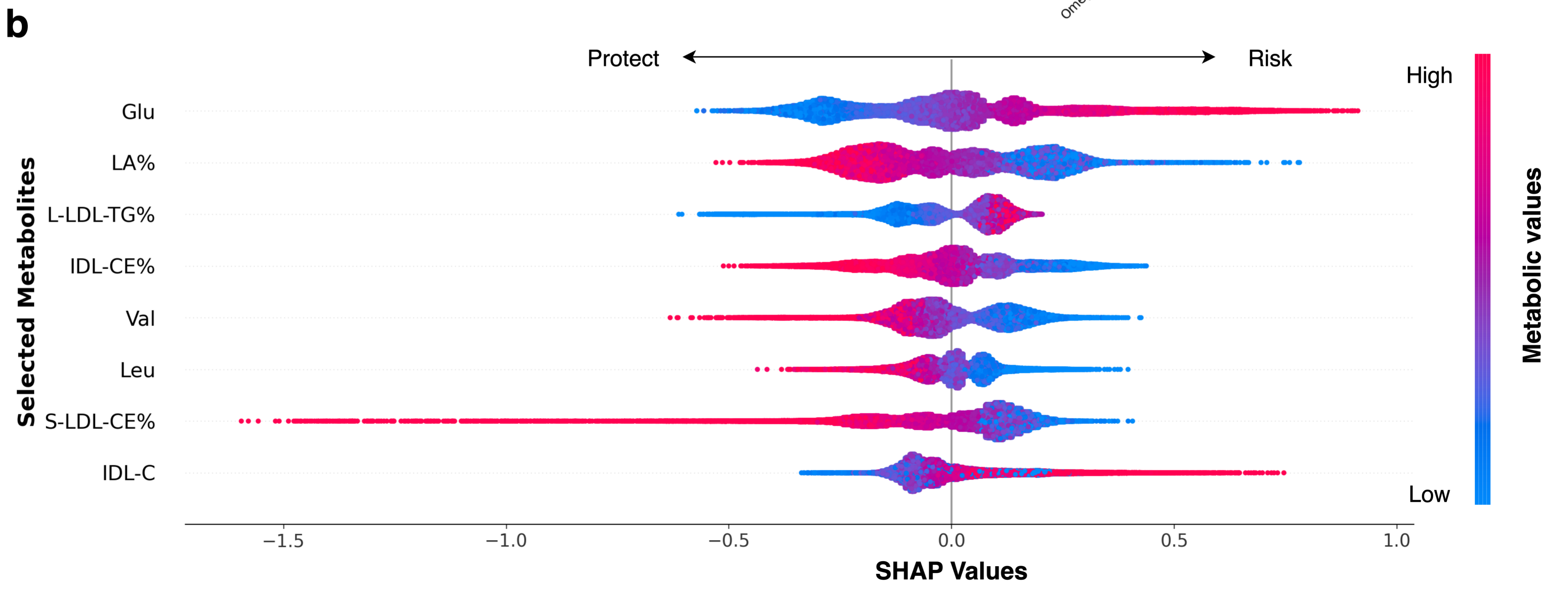

Supplement: Supplementary file 2 — Additional file 2: Supplemental Fig. 1. Selected metabolites to construct the MetRS of AD [file 13195_2023_1379_MOESM2_ESM.pdf]

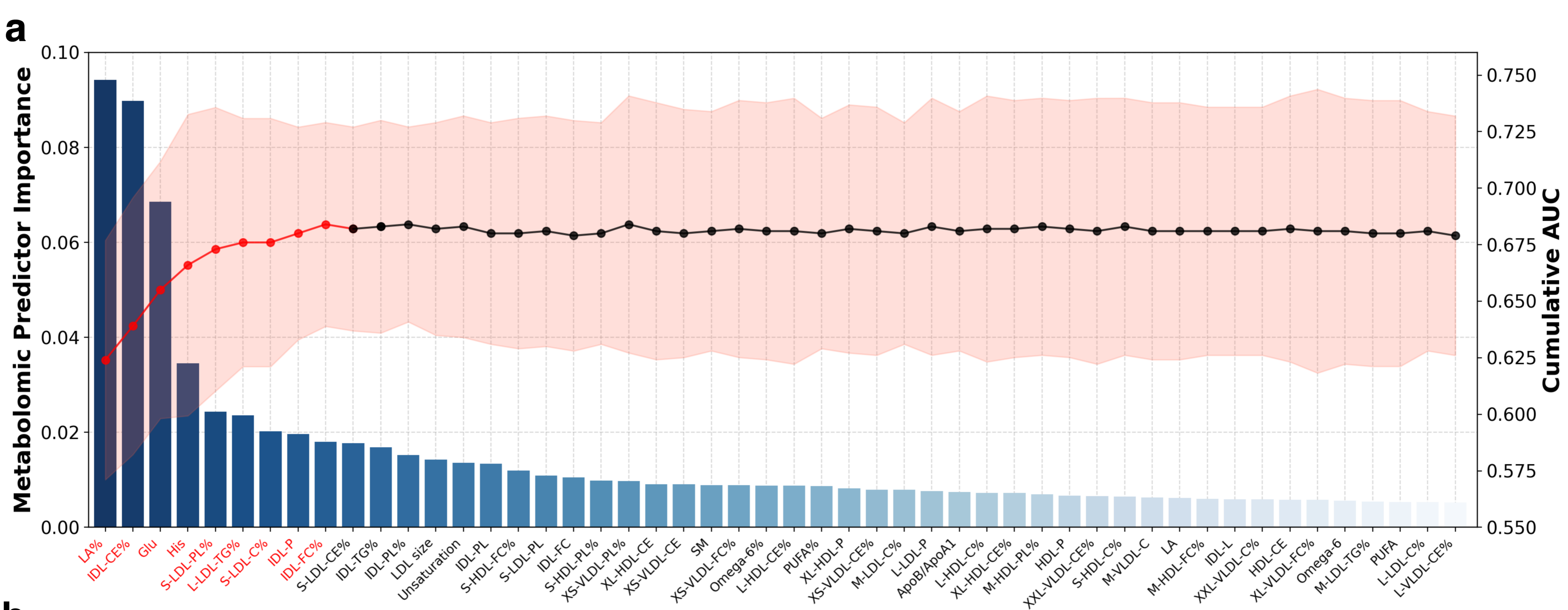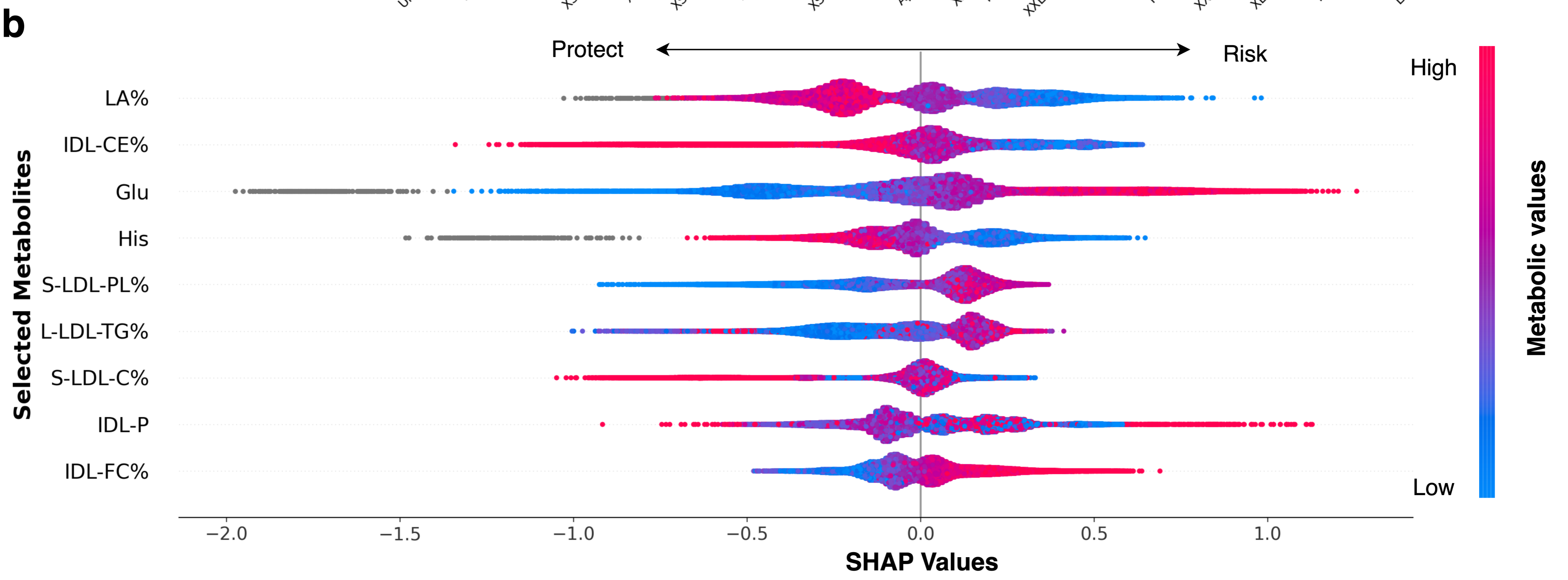

Supplement: Supplementary file 3 — Additional file 3: Supplemental Fig. 2. Selected metabolites to construct the MetRS of VaD [file 13195_2023_1379_MOESM3_ESM.pdf]

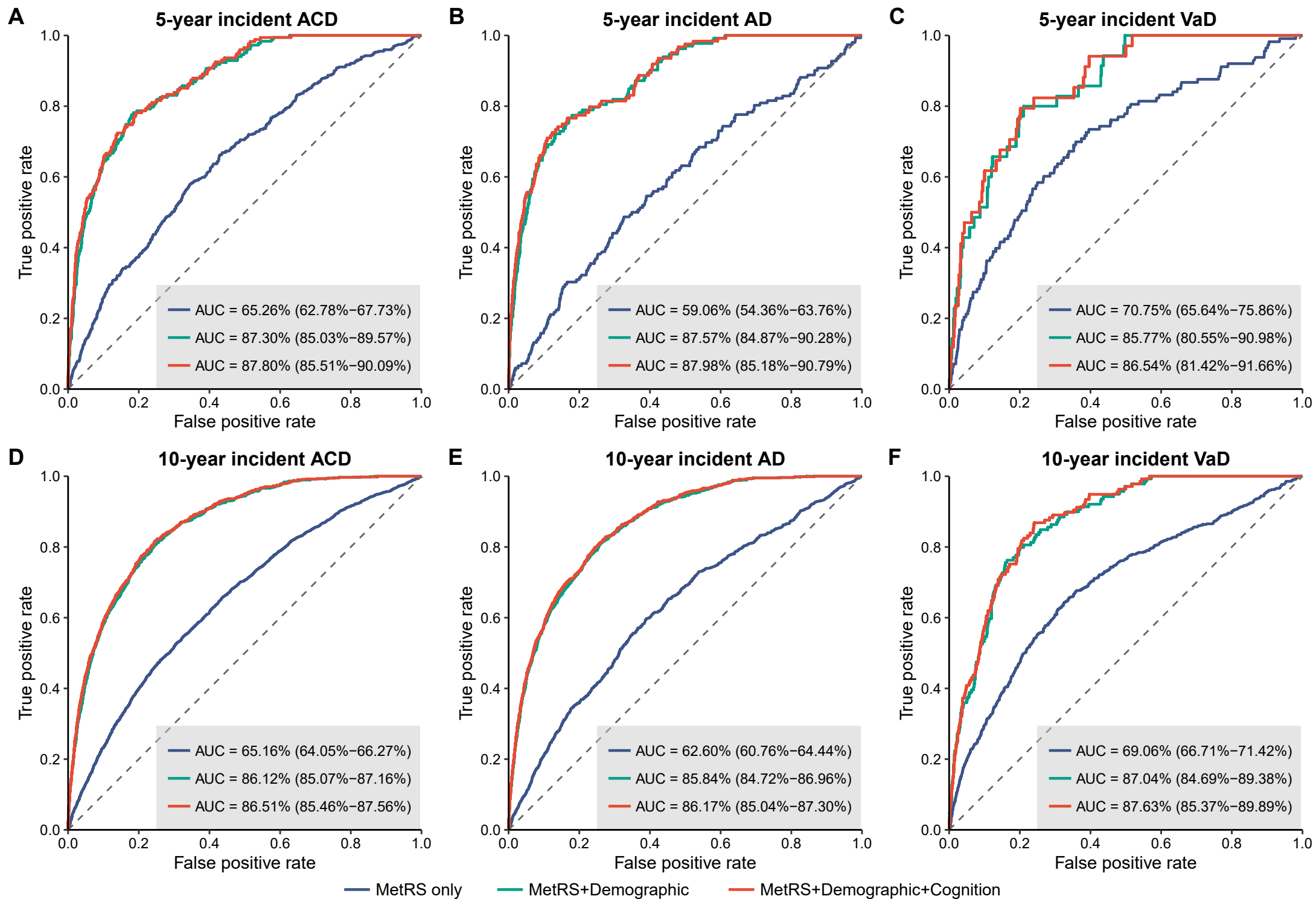

Supplement: Supplementary file 4 — Additional file 4: Supplemental Fig. 3. Results from utilizing the models for predicting 5-year, 10-year, and over 10-year probability of dementia [file 13195_2023_1379_MOESM4_ESM.pdf]
